# Resected thymic large cell neuroendocrine carcinoma: A case report and review of the literature

**DOI:** 10.1016/j.ijscr.2019.05.057

**Published:** 2019-06-08

**Authors:** Shogo Ogata, Ryo Maeda, Masaki Tomita, Yuichiro Sato, Takanori Ayabe, Kunihide Nakamura

**Affiliations:** a Department of Thoracic and Breast Surgery, University of Miyazaki, Miyazaki, Japan; b Department of Pathology, University of Miyazaki, Miyazaki, Japan; c Department of Cardiovascular Surgery, University of Miyazaki, Miyazaki, Japan

**Keywords:** Large cell neuroendocrine carcinoma, Thymus, Surgery, Thymic carcinoma

## Abstract

**Introduction:**

Large cell neuroendocrine carcinoma (LCNEC) of the thymus is an extremely rare neoplasm.

**Presentation of case:**

We report a rare case of LCNEC of the thymus in a 55-year-old woman. Her chest roentgenogram during a routine checkup revealed an abnormal shadow in the mediastinal left upper lung field. Chest computed tomography showed an anterior mediastinal mass measuring 4.8 × 4.0 cm. Positron emission tomography with 18F-fluorodeoxyglucose (FDG) showed high FDG accumulation at the lesion. To obtain a definitive diagnosis and achieve complete resection, a surgery was performed. The postoperative diagnosis was thymic LCNEC; it was classified as a Masaoka stage III tumor due to the invasion of tumor cells into the left lung. Postoperatively, the patient received adjuvant chemotherapy and survived without any signs of recurrence for 30 months after surgery.

**Discussion/conclusion:**

The detailed clinical features of thymic LCNEC remain unknown because of its rarity. In total, 20 cases of resection for LCNEC, including the present case, have been reported in the English language literature; we have presented a review of these cases and discussed the optimal therapy for this rare and virulent tumor of the thymus.

## Highlights

• Large cell neuroendocrine carcinoma (LCNEC) of the thymus is an extremely rare neoplasm and has a poor prognosis.
• We report a surgical case of thymic LCNEC.
• Further accumulation of knowledge and experience is needed to elucidate the optimal therapy for thymic LCNEC.

## 1 Introduction

The following case report has been reported from Our University Hospital which is an internationally recognized teaching hospital and a tertiary care centre, in accordance with the SCARE guidelines for case reports [1]. Large cell neuroendocrine carcinoma (LCNEC) of the thymus is a high-grade thymic tumor composed of large cells with neuroendocrine morphology and either neurosecretory granules on electron microscopy or positive neuroendocrine immunohistochemical markers [[2], [3], [4]]. Furthermore, thymic LCNEC is an extremely rare neoplasm and has a poor prognosis [2]. Because of its rarity, the detailed clinical features of thymic LCNEC remain unknown and no standard treatment has been established. In this report, we describe a surgical case of thymic LCNEC and review the cases of resection reported in the English language literature. Furthermore, we discuss the optimal therapy for this rare tumor of the thymus.

## 2 Presentation of case

**Fig. 1:**
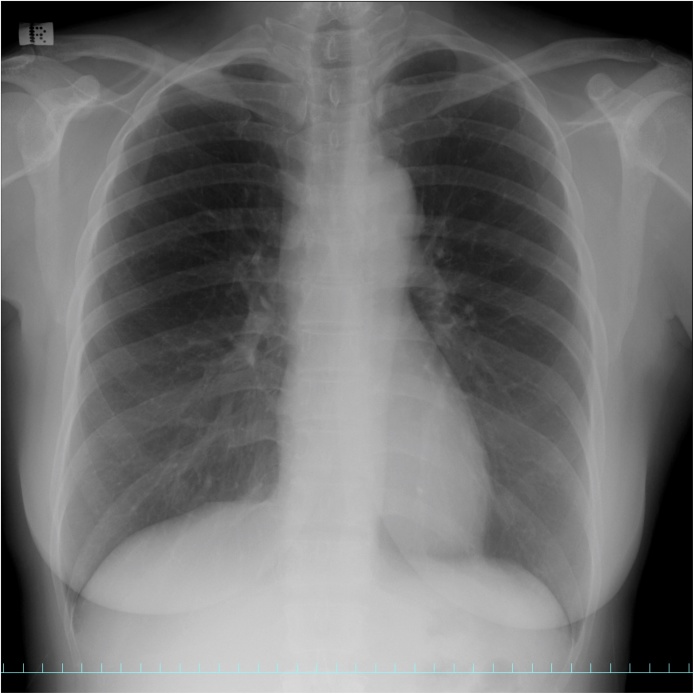
A computed tomographic scan of the chest shows an anterior mediastinal mass.

**Fig. 2:**
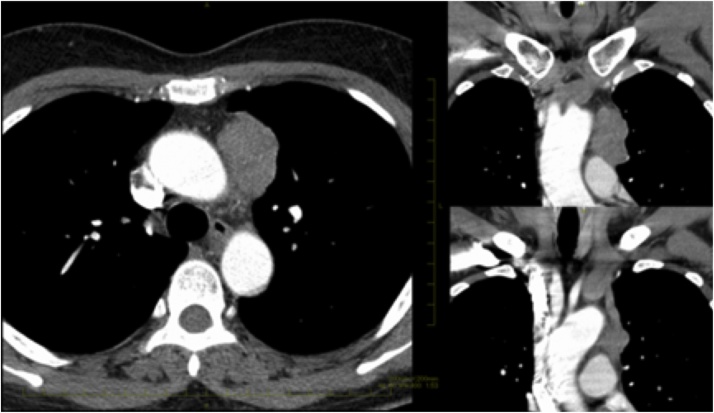
A computed tomographic scan of the chest shows an anterior mediastinal mass.

**Fig. 3:**
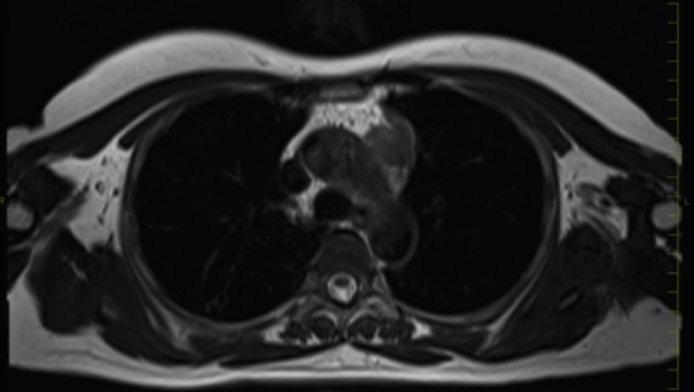
Magnetic resonance imaging of the chest shows high intensity on T2-weighted images with suspected invasion to the upper lobe of the left lung.

**Fig. 4:**
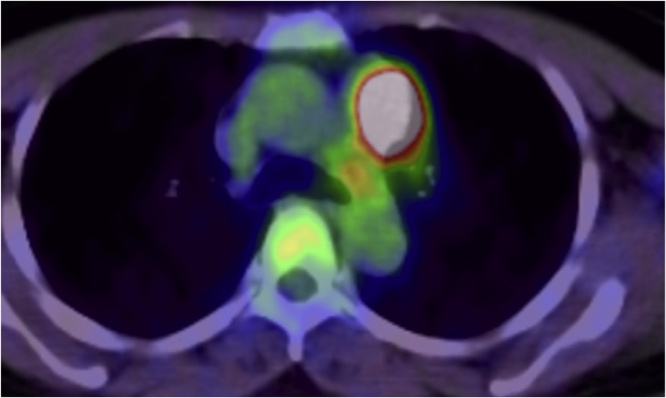
Positron emission tomography with 18F-fluorodeoxyglucose shows high 18F-fluorodeoxyglucose accumulation at the lesion.

An asymptomatic 55-year-old woman underwent a chest roentgenogram during a routine checkup; it showed an abnormal shadow in the mediastinal left upper lung field (Fig. 1). Chest computed tomography images showed a well-defined anterior mediastinal mass measuring 4.8 × 4.0 cm (Fig. 2). Tumor markers including carcinoembryonic antigen, cytokeratin fragment 21, progastrin-releasing peptide, α-fetoprotein, and human chorionic gonadotropin were found to be within the normal range. Chest magnetic resonance imaging showed an iso-intensity mass on T1-weighted images and high-intensity mass on T2-weighted images, with suspected invasion to the upper lobe of the left lung (Fig. 3). Positron emission tomography (PET) with ^18^F-fluorodeoxyglucose (FDG) showed high FDG accumulation at the lesion [maximum standardized uptake value (SUV_max_) of 12.75] (Fig. 4). To avoid the incidence of tumor cell implantation and pleural recurrence after needle biopsy, we did not perform preoperative percutaneous needle biopsy. Because a malignant tumor was suspected and complete resection of the tumor was considered possible, surgical removal of the tumor through median sternotomy was performed to obtain a definitive diagnosis and achieve complete resection. Because of tumor invasion, partial resection of the left upper lobe was performed. The tumor involved the left phrenic nerve; thus, the nerve was also resected.

**Fig. 5:**
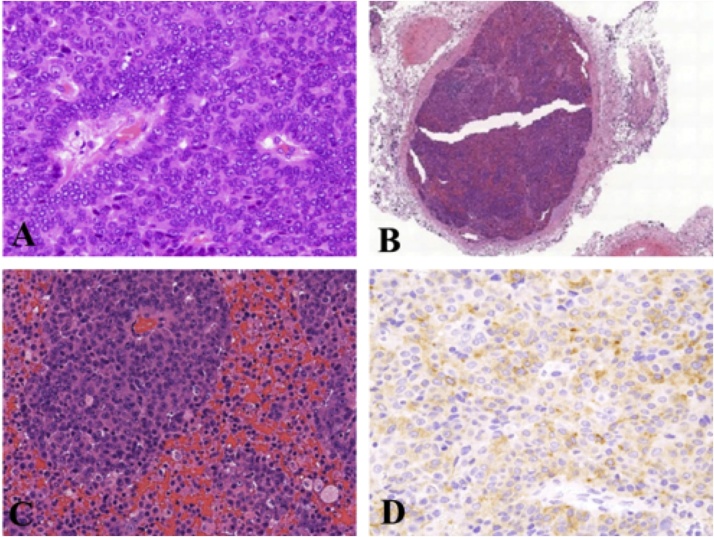
(A) The tumor cells with hyperchromatic nuclei proliferatein layered nest pattern with peripheral palisading and necrosis. Tumor embolism of the left innominate vein (B) or venous permeation in the tumor (C) are found. (D) Tumor cells are immunohistochemically positive for synaptophysin.

Histopathological examination of the tumor specimen revealed tumor cells with hyperchromatic nuclei proliferating in a layered nest pattern with peripheral palisading and necrosis (Fig. 5A). Tumor emboli (Fig. 5B) or venous permeation (Fig. 5C) were frequently seen. Immunohistochemically, the tumor cells were positive for synaptophysin (Fig. 5D) and chromogranin A, but negative for cytokeratin 5/6. The postoperative histopathological diagnosis was thymic LCNEC; it was classified as a Masaoka stage III tumor due to the invasion of tumor cells into the left upper lobe. Postoperatively, the patient received adjuvant chemotherapy (4 courses of cisplatin + etoposide). She survived without any signs of recurrence for 30 months after surgery.

## 3 Discussion

The thymus is one of the rarest sites for occurrence of neuroendocrine tumors (NETs) (first described by Rosai and Higa in 1972 [3]), with NETs of the thymus accounting for only 2%–5% of all thymic neoplasms [4]. The latest World Health Organization thymic epithelial tumor criteria^3^ have classified NECs as a subtype of thymic carcinoma. Furthermore, NETs have been classified as low grade for typical carcinoids, intermediate grade for atypical carcinoids, and high-grade for LCNEC and small cell carcinomas [3]. The majority of thymic NETs are carcinoid tumors, with LCNECs account for only 14%–26% of all thymic NETs [4].

Moran et al. [5] have reported that 5-year survival rates of patients with thymic NETs were 50%, 20%, and 0% for low-grade, intermediate-grade, and high-grade tumors, respectively. In the present case, FDG-PET showed a high SUV_max_. Considering the high SUV_max_ and poor prognosis for high-grade NETs, thymic LCNECs were thought to have high malignant potential, likely representing thymic carcinoma. However, thymic LCNECs are extremely rare, as described above; thus, their detailed clinical features remain unknown.

To date, 19 cases of resection for thymic LCNEC have been reported in the English language literature [[6], [7], [8], [9], [10], [11], [12], [13], [14], [15]]. The author summarized a total of 20 patients, including the present case, with ages between 42 and 90 (mean age, 57) years (Table 1). Of the 20 patients, 12 (60%) were male, and the tumor size ranged from 31 to 170 mm. The tumors were classified as Masaoka stage I in 1, stage II in 1, stage III in 11, stage IVa in 3, and with stage IVb in 4 patients. Of the 20 patients, 18 (90%) had advanced-stage tumors with stage III or higher.

**Table 1:** Reviews of twenty resected cases of thymic LCNEC in the English language literature.

Number	Year	Author	Age	Sex	Size (mm)	Preoperative therapy	Postoperative therapy	Masaoka stage	Recurrence (months)	Recurrence site	Observation (months)
1	2003	Tiffert et al. [6]	75	M	170	None	Radiotherapy	III	No		67
2	2006	Nagata et al. [7]	57	F	70	None	Chemoradiotherapy	III	7	Lung	11
3	2008	Mega et al. [8]	67	F	50	None	Chemoradiotherapy	IVb	6	Brain, Bone	9
4	2009	Dutta [9]	44	M	80	None	Chemoradiotherapy	III	6	Bone	13
5	2010	Cardillo et al. [10]	48	M	Not available	Chemotherapy	Radiotherapy	III	No		73
6	2010	Cardillo [10]	49	M	Not available	Chemoradiotherapy	Radiotherapy	III	No		69
7	2010	Cardillo et al. [10]	50	F	Not available	not available	Radiotherapy	IVa	No		51
8	2010	Cardillo [10]	48	F	Not available	not available	Radiotherapy	III	No		13
9	2010	Cardillo et al. [10]	46	M	Not available	not available	Radiotherapy	III	No		95
10	2012	Ahn et al. [11]	67	M	90	Radiotherapy	Chemoradiotherapy	IVb	3	Local recurrence	3
11	2012	Ahn [11]	42	M	85	Chemoradiotherapy	None	III	1	Bone	7
12	2012	Ahn et al. [11]	72	F	73	Chemotherapy	Radiotherapy	IVa	2	Bone, Liver, Mediastinum	4
13	2012	Yoon et al. [12]	64	M	85	None	None	IVb	9	Liver, Adrenal gland, Bone	48
14	2012	Yoon et al. [12]	57	M	170	Chemotherapy	Radiotherapy	IVb	12	Bone	12
15	2015	Igawa et al. [13]	59	M	40	None	Chemotherapy	II	48	Pleural dissemination, Mediastinal lymphnode	61
16	2018	Domen et al. [14]	90	M	31	None	None	III	No		12
17	2018	Ose et al. [15]	80	F	65	None	Not available	IVa	10	Lung	71
18	2018	Ose [15]	57	F	90	None	Radiotherapy	I	No		30
19	2018	Ose et al. [15]	44	M	78	Chemoradiotherapy	Not available	III	10	Bone	64
20	2019	Our case	55	F	48	None	Chemotherapy	III	No		30

Of the 20 patients, 11 (55%) developed recurrence. Distant metastases were found in 9 of 11 patients. In the present case, venous permeation or tumor emboli were frequently seen in the resected specimen. In patients with lung cancer, intra-tumoral vascular invasion indicates postoperative distant metastases [16]. Because complete resection reportedly contributes to good prognosis in patients with thymic cancer [17], surgery should be recommended in patients with thymic LCNEC as well; however, surgery alone may be insufficient in such cases because of the high frequency of postoperative distant metastases.

Several studies on LCNEC of the lung have recommended postoperative administration of adjuvant chemotherapy with platinum-based combination regimens, which are used for small cell lung carcinoma that exhibits clinicopathological and biological features similar to LCNEC [18,19]. Likewise, we believe that surgery and adjuvant chemotherapy are needed to treat thymic LCNEC, even in the cases of complete resection. Although there is no evidence to support adjuvant therapy for thymic LCNEC, a regimen comprising cisplatin/carboplatin/etoposide (as for small cell lung carcinoma) seems the most common choice at present for thymic LCNEC. Therefore, adjuvant chemotherapy (4 courses of cisplatin + etoposide) was administered to our patient, and she survived without any signs of recurrence for 30 months after surgery. An effective therapeutic modality, combined with surgery, should be evaluated, and further studies are needed to elucidate the optimal therapy for this rare and virulent tumor of the thymus.

## 4 Conclusion

We reported a case of resection for thymic LCNEC. Thymic LCNEC is a highly virulent tumor of the thymus. Further accumulation of knowledge and experience is needed to elucidate the optimal therapy for this rare tumor of the thymus.

## Conflict of interest

There is no conflict of interest for any of the authors.

## Sources of funding

The authors state that the case report was produced in the absence of economic funding sources.

## Ethical approval

Ethical approval was not required from my Institution for this case report.

## Consent

Written informed consent was obtained from the patient for publication of this case report and accompanying images. A copy of the written consent is available for review by Editor-in-Chief of this journal on request.

## Author’s contribution

Shogo Ogata and Ryo Maeda conceptualised the study, performed a literature review and drafted the manuscript.

Ryo Maeda, Masaki Tomita, Takanori Ayabe and Kunihide Nakamura performed a literature review and drafted the manuscript.

Yuichiro Sato performed a literature review and collected pathological data.

Shogo Ogata, Ryo Maeda and Masaki Tomita performed a literature review and collected data.

Shogo Ogata, Ryo Maeda and Masaki Tomita critically revised the article.

All authors approved submission of the final article.

## Registration of research studies

Not applicable.

## Guarantor

Masaki Tomita.

## Provenance and peer review

Not commissioned, externally peer-reviewed.
